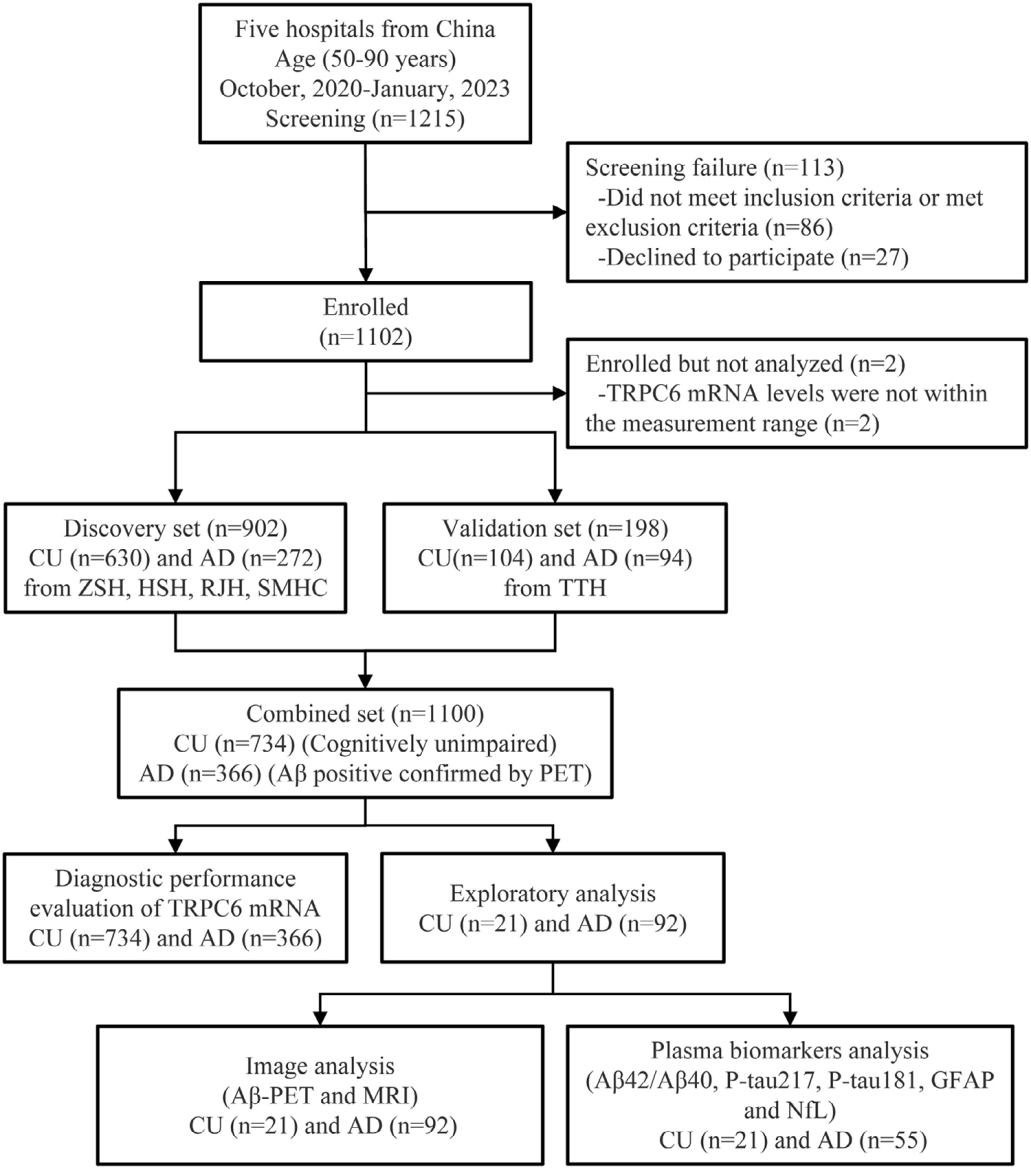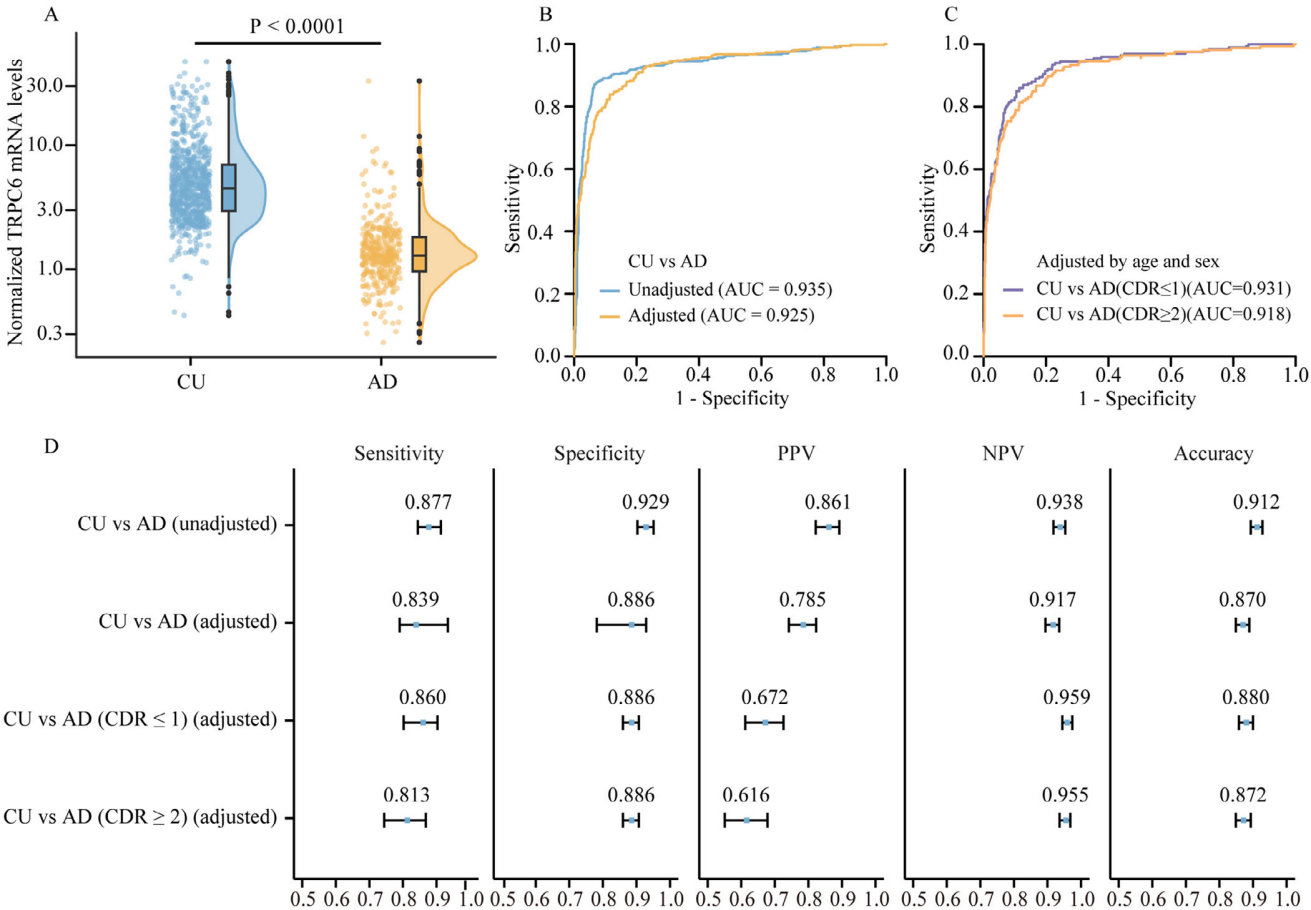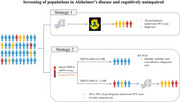# Blood TRPC6 mRNA as a Biomarker for Diagnosing Alzheimer's Disease: A Nationwide, Multicentre Trial in China

**DOI:** 10.1002/alz70856_098827

**Published:** 2025-12-24

**Authors:** Mei Pu, Qianhua Zhao

**Affiliations:** ^1^ Fudan University, Shanghai, Shanghai, China; ^2^ Institute for Translational Brain Research, State Key Laboratory of Medical Neurobiology, MOE Frontiers Center for Brain Science, Fudan University, Shanghai, Shanghai, China; ^3^ National Clinical Research Center for Aging and Medicine, Huashan Hospital, Fudan University, Shanghai, Shanghai, China; ^4^ Huashan Hospital, Fudan University, Shanghai, Shanghai, China; ^5^ National Center for Neurological Disorders, Huashan Hospital, Fudan University, Shanghai, China

## Abstract

**Background:**

Diagnosis of Alzheimer's disease (AD) generally rely on either positron emission tomography (PET) imaging or cerebrospinal fluid (CSF) testing for β‐amyloid (Aβ) and tau proteins, which are expensive and invasive. Finding biomarkers with better accessibility and lower cost remains a critical challenge in the screening and diagnosis of AD. We investigated whether blood transient receptor potential canonical 6 (TRPC6) protein, a molecule essential for synaptogenesis and neuronal survival, could serve as a reliable diagnostic biomarker for AD.

**Method:**

A total of 1,100 individuals (*n* = 1,100) were enrolled, including AD (*n* = 366) patients and cognitively unimpaired (CU) individuals (*n* = 734). Aβ deposition was determined by ^18^F‐flobetapir PET, while brain volume was quantified via magnetic resonance imaging (MRI). All study participants underwent TRPC6 mRNA assessments. AD patients were confirmed as Aβ positive through PET imaging. A subset of participants underwent Aβ‐PET imaging, 3‐dimensional (3D) T1‐weighted MRI, and plasma biomarkers analysis.

**Result:**

The median age of 1,100 participants was 68.67 years (IQR: 63.72 to 73.91) with women participants accounted for (60.18%). Among the 366 AD patients, 200 were mild AD (CDR≤1), 166 were moderate and severe AD (CDR≥2). Blood TRPC6 mRNA levels were significantly lower in AD patients compared to CU individuals (1.29 vs. 4.50, *p* <0.0001). ROC curve analysis demonstrated that blood TRPC6 mRNA levels accurately identified AD patients from CU individuals, with an unadjusted area under the curve (AUC) of 0.935 (95% CI 0.917–0.952), and an adjusted AUC by age and sex of 0.925 (95% CI 0.908–0.943). Voxel‐wise analyses revealed a strong association between blood TRPC6 mRNA levels and both Aβ deposition and brain volume. Additionally, when comparing to other plasma AD biomarkers, TRPC6 mRNA levels demonstrated satisfactory diagnostic performance, surpassing plasma Aβ42/40, *p*‐tau181, GFAP and NfL, while being slightly inferior to *p*‐tau217 (TRPC6, AUC=0.935; Aβ42/40, AUC=0.879; *p*‐tau181, AUC=0.924; GFAP, AUC=0.894; NfL, AUC=0.734; *p*‐tau217, AUC=0.965).

**Conclusion:**

Blood TRPC6 mRNA levels exhibited excellent diagnostic utility in identifying patients with AD from CU individuals. As a blood biomarker, TRPC6 mRNA levels offer a feasible, scalable, and cost‐effective diagnostic tool that could serve as a standalone index for AD.